# The Lithic Assemblages of Xiaochangliang, Nihewan Basin: Implications for Early Pleistocene Hominin Behaviour in North China

**DOI:** 10.1371/journal.pone.0155793

**Published:** 2016-05-20

**Authors:** Shi-Xia Yang, Ya-Mei Hou, Jian-Ping Yue, Michael D. Petraglia, Cheng-Long Deng, Ri-Xiang Zhu

**Affiliations:** 1 State Key Laboratory of Lithospheric Evolution, Institute of Geology and Geophysics, Chinese Academy of Sciences, Beijing, China; 2 Key Laboratory of Vertebrate Evolution and Human Origin of Chinese Academy of Sciences, Institute of Vertebrate Paleontology and Paleoanthropology, Chinese Academy of Sciences, Beijing, China; 3 University of Chinese Academy of Sciences, Beijing, China; 4 School of Archaeology, University of Oxford, Oxford, United Kingdom; Universidade do Algarve, PORTUGAL

## Abstract

Xiaochangliang (XCL), located in the Nihewan Basin of North China, is a key archaeological locality for understanding the behavioural evolution of early humans. XCL dates to ca. 1.36 Ma, making it one of the earliest sites in Northeast Asia. Although XCL represents the first excavation of an Early Pleistocene site in the Nihewan Basin, identified and excavated in the 1970’s, the lithic assemblages have never been published in full detail. Here we describe the lithic assemblages from XCL, providing information on stone tool reduction techniques and the influence of raw materials on artefact manufacture. The XCL hominins used both bipolar and freehand reduction techniques to manufacture small flakes, some of which show retouch. Bipolar reduction methods at XCL were used more frequently than previously recognized. Comparison of XCL with other Early Pleistocene sites in the Nihewan Basin indicates the variable use of bipolar and freehand reduction methods, thereby indicating a flexible approach in the utilization of raw materials. The stone tools from XCL and the Nihewan sites are classifiable as Mode I lithic assemblages, readily distinguished from bifacial industries manufactured by hominins in Eastern Asia by ca. 800 ka.

## Introduction

**Fig 1:**
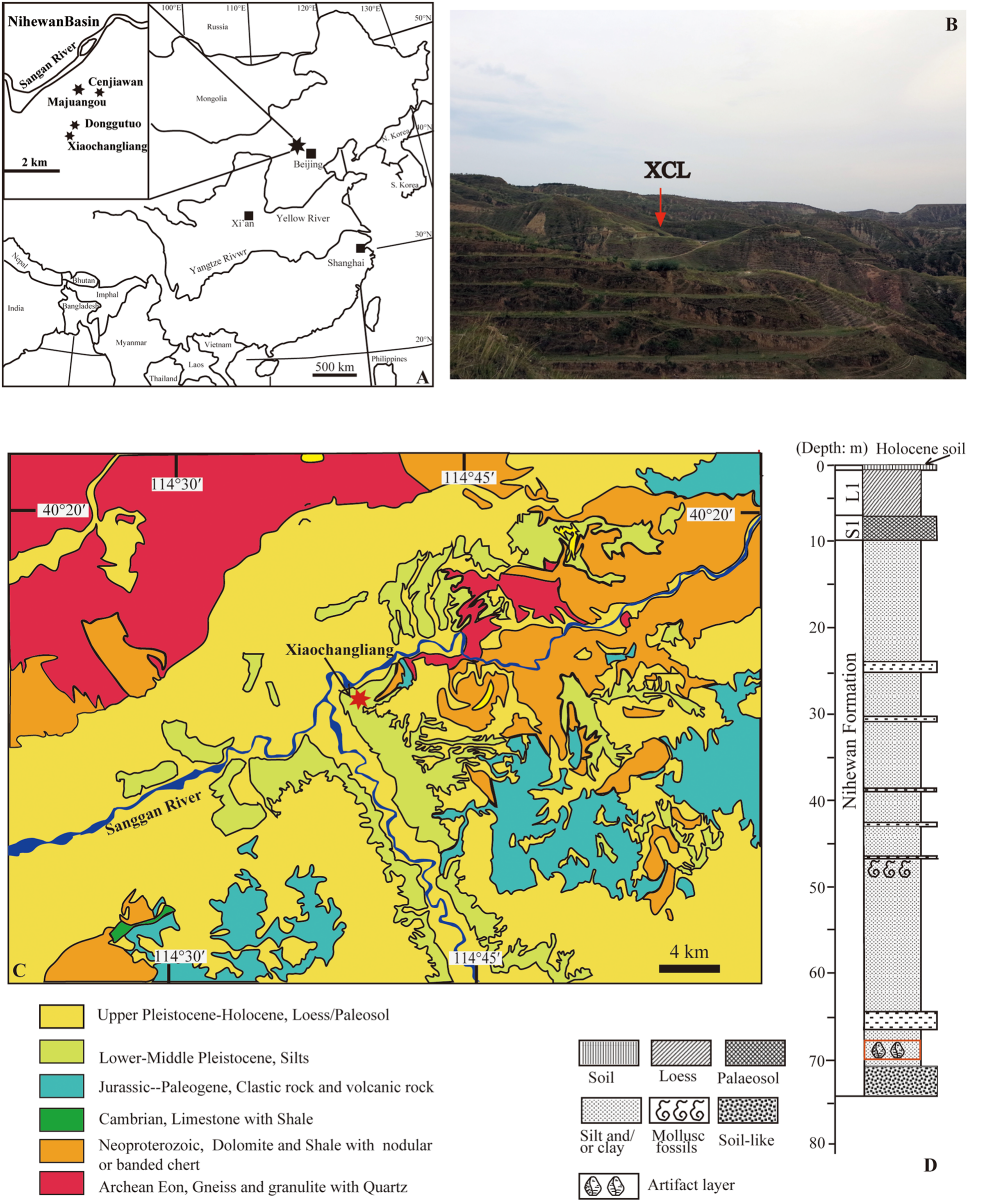
XCL, Nihewan Basin, China. (A) The Nihewan Basin, showing the location of Early Pleistocene sites; (B) The dissected terrain of the eastern Nihewan Basin showing the location of XCL; (C) Simplified geological map of the Nihewan Basin and raw material sources. Note that XCL occurs in Lower-Middle Pleistocene sedimentary deposits; (D) The general stratigraphy of XCL, showing the location of the artefact horizon in the Nihewan Formation.

The Nihewan Basin in North China is one of the most important palaeoanthropological archives in Asia as it preserves an abundance of mammal fossils and stone tool assemblages in a number of localities (e.g., Majuangou, Xiaochangliang, Huojiadi, Feiliang, Donggutuo, Cenjiawan) (Fig 1A–1C). The Early Pleistocene sites are dated to between the Gauss–Matuyama and Matuyama–Brunhes geomagnetic reversals (0.78–2.59 Ma) [1–4], thus representing some of the earliest archaeological sites in Eastern Asia. Although hominin fossils have not been recovered from the Nihewan Basin as of yet, early human activities are well represented by tens of thousands of stone tool artefacts [5].

Xiaochangliang (XCL) (40°13′10″N,114°39′44″E) was discovered in 1978 and later dated to ca. 1.36 Ma by magnetostratigraphy [1]. XCL was the first Early Pleistocene archaeological site discovered in the Nihewan Basin, and thus regarded as a milestone in human evolutionary studies in China, particularly for understanding the expansion of early hominins to high latitudes. Since its original discovery in 1978, a series of field investigations and archaeological excavations have been conducted at XCL, leading to the recovery of fossils and stone artefacts. Despite the importance of the site for understanding early human behaviour, the lithic assemblages have never been adequately described. Here we summarize available information about the XCL lithic assemblages and present new information about stone tool technology. Our main aim here is to evaluate stone tool reduction methods employed by Nihewan knappers and to situate this information into current discussions about the behaviour of early hominins in Eastern Asia.

## Field Setting

Xiaochangliang is located at the eastern edge of the Nihewan Basin (Fig 1A–1C). The Nihewan Basin spreads over the counties of Yangyuan and Yuxian, covering an area of roughly 150–200 km^2^. The Sanggan River meanders across the basin, which is filled with Late Pliocene to Holocene lacustrine, fluvial and aeolian deposits [1–4, 6, 7]. The fluvio-lacustrine sedimentary sequence below the wind-blown sediments are known as the Nihewan Beds [8]. The Nihewan Formation, which represents the type section of the Early Pleistocene in North China [9], was initially restricted to the lower portion of the Nihewan Beds. However, in recent studies the term “Nihewan Formation” has been used to define the whole fluvio-lacustrine sequence in the basin [3,6,10].

The XCL stone artefact layer is located along cliffs of the southern bank of the Sanggan River and lies approximately 66.5 m below the Holocene sediments (Fig 1D). The cultural deposits are in sediments ranging between 0.5–0.8 m in thickness. Palaeomagnetic dating demonstrated that the top of the artefact layer lies 13.4 m below the Jaramillo Subchron, and the age of the artefact layer was estimated to be ca. 1.36 Ma [1]. The age estimate was based on the assumed average sediment accumulation rates between the Jaramillo and Olduvai Subchrons. Others researchers, however, contend that the artefact layer may be somewhat older, i.e., ca. 1.48 Ma [11] or possibly younger, i.e. 1.26 Ma [12] than the original age estimate (for a review, see [13]). The deposits preserve a rich vertebrate faunal assemblage, including *Allophaiomys* cf. *A*. *pliocaenicus*, *Mimomys chinensis*, *Hyaena (Pachycrocuta) licenti*, *Palaeoloxodon* sp., *Hipparion* sp., *Proboscidipparian sinensis*, *Equus sanmeniensis*, *Coelodonta antiquitatis*, *Martes* sp., *Cervus* sp. and *Gazella* sp. The taxa are consistent with an Early Pleistocene age [14]. The fauna at XCL, in combination with other stratigraphically equivalent localities [15, 16], imply dominant grasslands with patches of woodlands.

## History of Research at XCL and Site Formation

Xiaochangliang was first excavated in 1978, and at that time, a total of 804 lithic artefacts was recovered from two site areas, though the artefacts were not provenienced in the field or well-described [17]. During 1990 to 1997, five excavation seasons were sponsored by the Institute of Vertebrate Paleontology and Paleoanthropology (IVPP) of the Chinese Academy of Sciences, and a further 1258 artefacts were collected, though none of the artefacts were provenienced as to excavation unit and level, and the results were not published. In 1998, an excavation was conducted by Fudan University, and 901 lithic artefacts were recovered and reported [18].

The 1998 excavations were systematic and employed GIS mapping, 3D piece plotting, digital photography, use-wear analysis and taphonomic studies [19]. The sediments of the artefact horizon were previously described as a grey silty sediment with yellowish sandy soils [17, 20]. The 1998 excavations clarified the nature of the sedimentary horizon housing the artefacts and fossils. Field observations indicated that the horizontal bands of the yellow sandy soil were circular, semicircular and lineal in plan view, and demarcated from the surrounding grey silty sediment. Plotting of lithics and fauna indicated that the positions of the materials were influenced by water flow [18, 19]. Small artefacts and bone fragments were sometimes vertically inclined and often at the outer margins of the instrusive yellow sands. Moreover, the ‘even’ horizontal distribution of small lithic artefacts (< 0.5 cm in length) across the excavation units suggested their spread by natural agencies across the cultural horizon. Hydrological movement was further suggested by the faunal elements, which showed evidence of surface abrasion [21]. The overall interpretation offered from the 1998 excavation was that the deposits were the consequence of primary hominin activity and buried soon after site abandonment, though artefacts and fossils were subject to short distance rearrangement by water flow [19, 21], a situation found in other Early Pleistocene sites located elsewhere [22].

## Results

### Raw material selection

**Fig 2:**
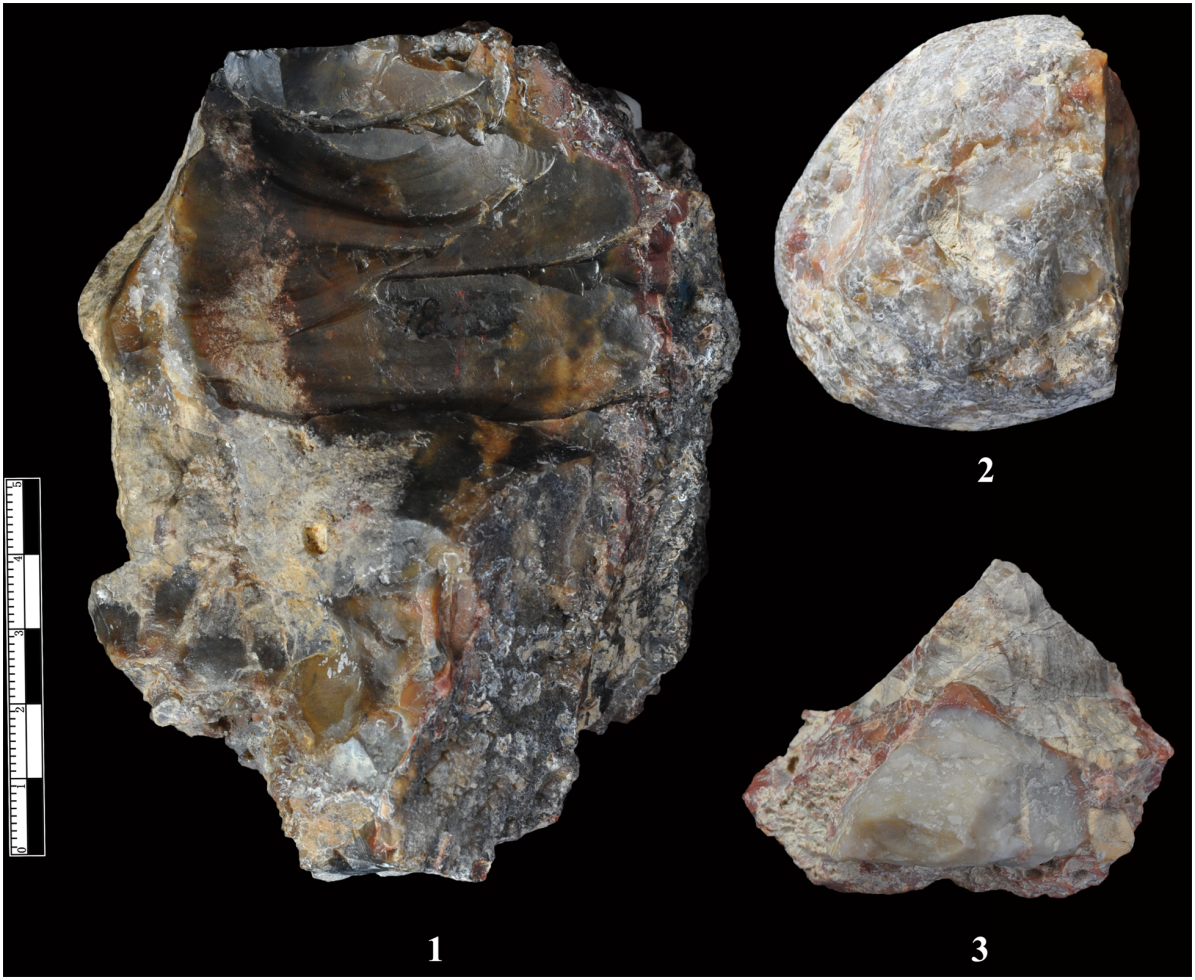
Lithic raw material quality. The artefacts show that some areas are fine-grained chert, while on other parts of the same piece, interstitial material and other kinds of breccia are preserved.

Inspection of the lithic artefacts located at the IVPP indicated that the XCL hominins mainly selected chert of varying colours (96.4%) for stone tool reduction, though low numbers of volcanic rock, quartz and limestone were present. The chert artefacts have two possible sources, one from a nodular or bedded outcrop and the other from pyroclastic rocks, mainly with asymmetric and sub-angular breccias of chert, dolomite, limestone and quartzite. Some of the chert artefacts retained interstitial material or breccia (Fig 2), indicating that pyroclastic forms were utilized.

According to geological maps (1:200,000 scale), the raw materials used for artefact manufacture (i.e., chert, quartz, volcanic rocks) can be found in the vicinity of XCL and in deposits within 3 km (Fig 1C). The nodular or bedded chert occurs in Neoproterozoic strata and the pyroclastic rocks containing chert breccias are found in Jurassic-Paleogene pyroclastic rocks (Fig 1C). Recent field inspection of XCL indicates that rock outcrops with chert can be found within 100–200 m from the site, thus indicating that raw materials were readily available to hominins.

**Fig 3:**
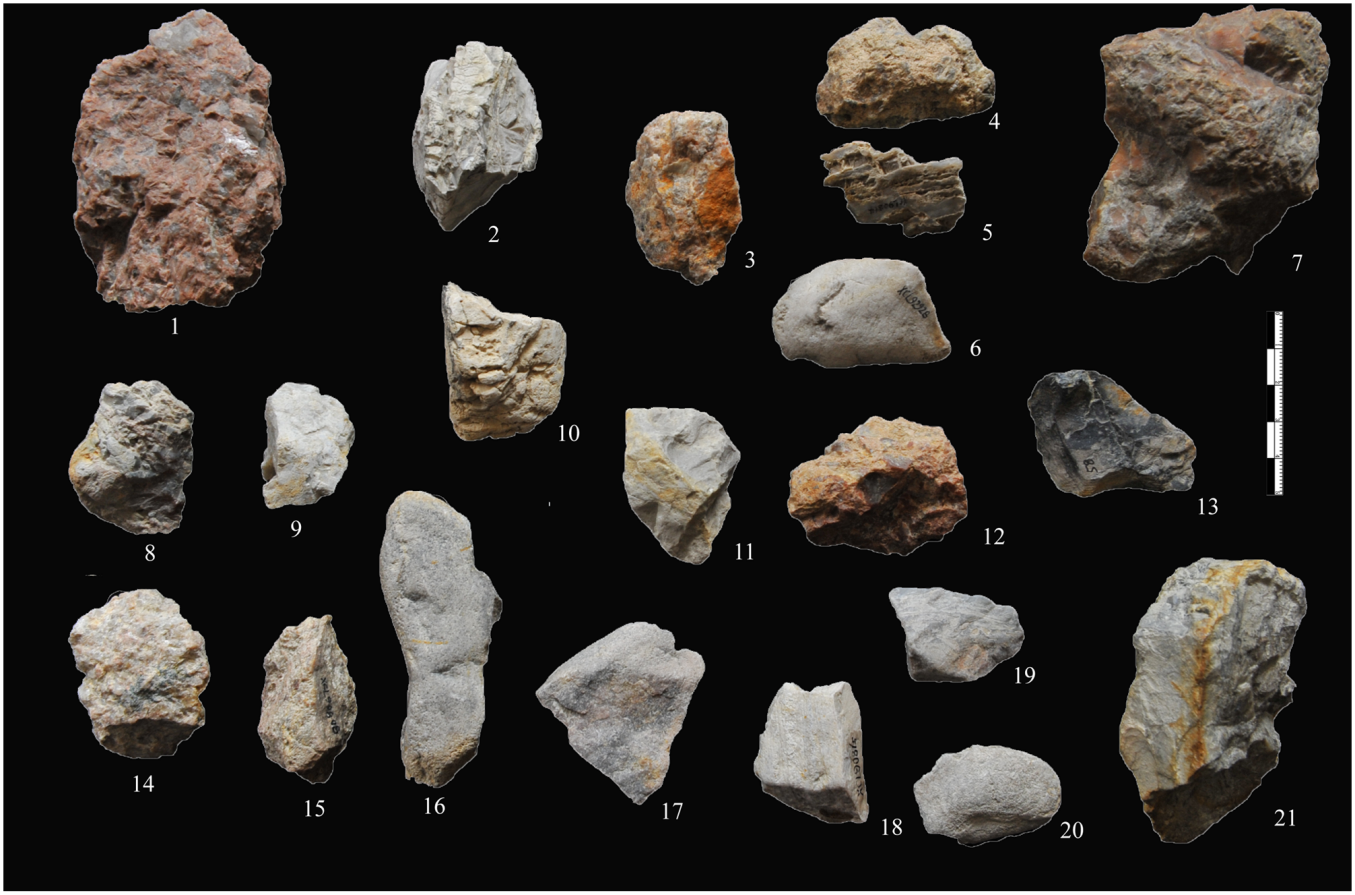
Unmodified materials from XCL. Most of the unmodified materials are small, coarse-grained, and irregular in shape.

Observations of the XCL lithic artefacts indicated the presence of internal flaws in the cherts. As a consequence, many knapping errors and unpredictable angular fragments were produced during percussion, as will be described below. Fine and homogeneous raw materials often occurred as small chert nodules, thus the bipolar technique was an effective solution for obtaining sharp-edged implements. Though previous studies have identified chert nodules on-site, suggesting that these were transported by hominins (Fig 3), their small size (92% are smaller than 40 mm), together with evidence of water flow, would suggest natural agencies may be responsible for their introduction, thus we have labeled them as unmodified materials in our study.

### XCL lithic assemblage

A total of 1468 lithic artefacts, stored in the IVPP, and recovered from the 1978 and 1990 to 1997 field seasons, was analyzed in this study. The 1998 field season report [18], comprising an assemblage of 901 lithic artefacts provided an additional source of information about the XCL lithic assemblages. While studies of the XCL lithic assemblages have been previously reported [18, 23], the current study represents a more comprehensive and detailed study, including a comparative analysis of Early Pleistocene lithic assemblages in the Nihewan Basin.

Table 1 is a compilation of data from previous lithic studies [17–19] in comparison to the present study. It appears that the 1978 study is a biased sample of lithic artefacts as the full range of lithic classes is not represented, and only includes a very high number and percentage of unclassified pieces (i.e., 720 chunks/shatter/angular), and a generally lower number of flaked artefacts in comparison to later studies.

**Table 1:** Lithic classifications of XCL artefacts according to previous studies and the current study.

Lithic class	1978^a^	%	1990–1997^b^	%	1998^b^	%	Current Study^c^	%
Core (Freehand)	25	3.11	26	2.07	39	4.33	43	2.82
Core (Bipolar)	--	--	59	4.69	27	3.00	111	7.27
Core fragment	--	--	18	1.43	5	0.55	--	--
Flake (Freehand)	47	5.85	232	18.44	143	15.87	160	10.49
Flake fragment	--	--	--	--	--	--	129	8.45
Splinter (Bipolar)	--	--	111	8.82	29	3.22	328	21.49
Retouched pieces (tools)	12	1.49	12	0.95	7	0.78	45	2.95
Nodules	--	--	31	2.46	40	4.44	--	--
Unmodified materials	--	--	--	--	--	---	340	22.28
Chunk/shatter/angular fragment	720	89.55	558	44.36	414	45.95	370	24.25
Chip	--	--	211	16.77	197	21.86	--	--
TOTAL	804	100	1258	100	901	100	1468	100

Note:

^a^[17]

^b^[19, 23]

^c^1978, 1990–97 collections

Chunks are rock fragments showing visible anthropic traces but which cannot be classified as flakes, flake fragments or cores. Nodules and Unmodified materials show no percussion or flaking evidence and thus are natural clasts.

Table 1 shows that the later lithic studies, performed in the 1990s, are in general agreement with respect to the proportions of cores produced by freehand reduction, ranging between 2–4% of the lithic assemblage. The freehand flake byproducts vary somewhat, totaling to between 18.44% and 15.87% in previous studies, and 10.49% in the present study. A main difference found between the previous studies and the current analysis is in the higher frequency of bipolar products. When first reported in 1978, no bipolar-related artefacts were recognized. In follow-on studies, bipolar artefacts were noted, though technological details were not provided [24, 25]. In the more systematic studies, bipolar products accounted for a lower proportion of the lithic assemblages, between 6.22% and 13.51% (Table 1). In the current study, bipolar products account for a much larger proportion of the lithic assemblage, totaling to 30.23%.

In earlier studies, bipolar flakes were only defined by the presence of double bulbs of percussion or battering scars on two edges. Yet, experimental studies have shown that bipolar reduction results in a range of other attributes, such as by battering on one or two edges, opposed scars, torsioned ventral surfaces and linear/point striking areas. Bipolar attributes recorded on the positives largely depend on the type and quality of the selected raw material and the type of bipolar reduction carried out [26–29]. As a consequence, many bipolar positives may be under-represented in earlier studies, being classified as “debris”, “chips” and “chunks” [23].

In our view, many of the small pieces without conchoidal percussion features are related to bipolar methods, especially given the presence of a large number and percentage of bipolar cores in our study (n = 111, 7.27%). If “chips” in the 1998 assemblage are considered as by-products of the bipolar technique, up to 30% of the lithic assemblage may be related to bipolar flaking methods. Moreover, a high percentage of “chunks” (n = 414, or 45.9%) in the 1998 assemblage [17, 18, 23] are also likely the consequence of bipolar methods. In our study we find a relatively high number and percentage (n = 370, 24.25%) of chunks and debris, likely relating to the identification of bipolar cores and splinters in the assemblage.

In sum, some significant differences are found between the current study and previous studies. While freehand and bipolar methods have been identified in multiple independent studies of XCL, we find that the bipolar method played a more important role than heretofore realized.

### Knapping methods

Freehand percussion and bipolar reduction methods identified at XCL are described below. Though recent studies show that the two methods are sometimes difficult to distinguish, a combination of qualitative and quantitative methods is considered the best approach [27, 30–32]. Accordingly, the following sections describe reduction methods and lithic artefact types from XCL.

#### Freehand reduction

A total of 43 cores, 160 flakes, and 129 flake fragments and splinters are identified as the product of freehand percussion methods. Here we place various types of flake fragments (e.g., “siret” flakes with broken platforms) into a single simplified category. Raw materials used for artefact production include chert, quartz and basalt, though chert predominates, representing 96.1% (n = 319) of the total assemblage.

**Fig 4:**
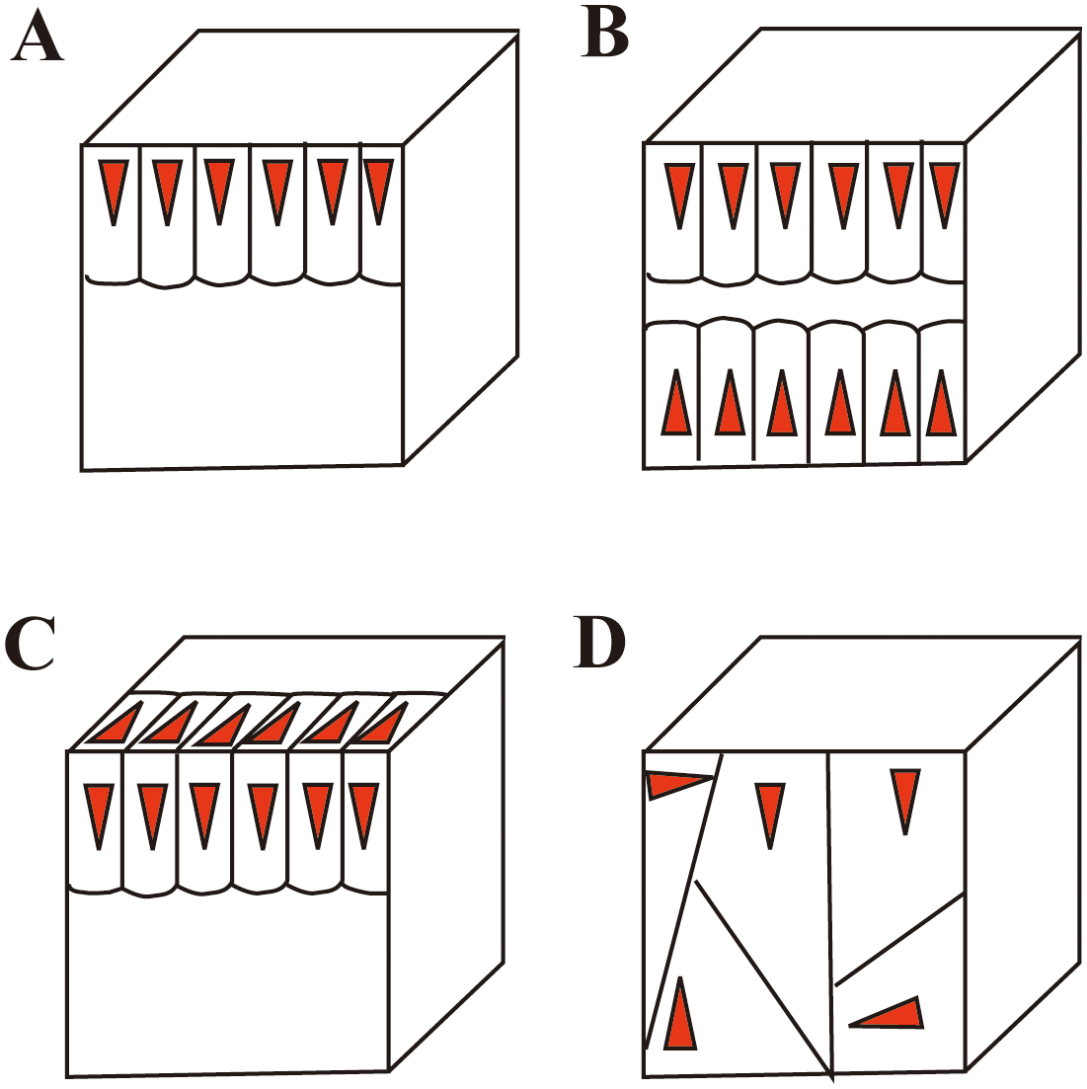
Idealized schemes of freehand cores. Type A: Simple and partial exploitation method, with unidirectional flaking and restricted to one plane on a natural striking platform. Type B: Bidirectional method, with simple and partial exploitation from two opposed platforms. Type C: Bifacial method with simple partial exploitation, with unidirectional flaking, but employed on two adjacent surfaces. Type D: Multifacial, multidirectional and irregular flaking method, from various directions.

**Fig 5:**
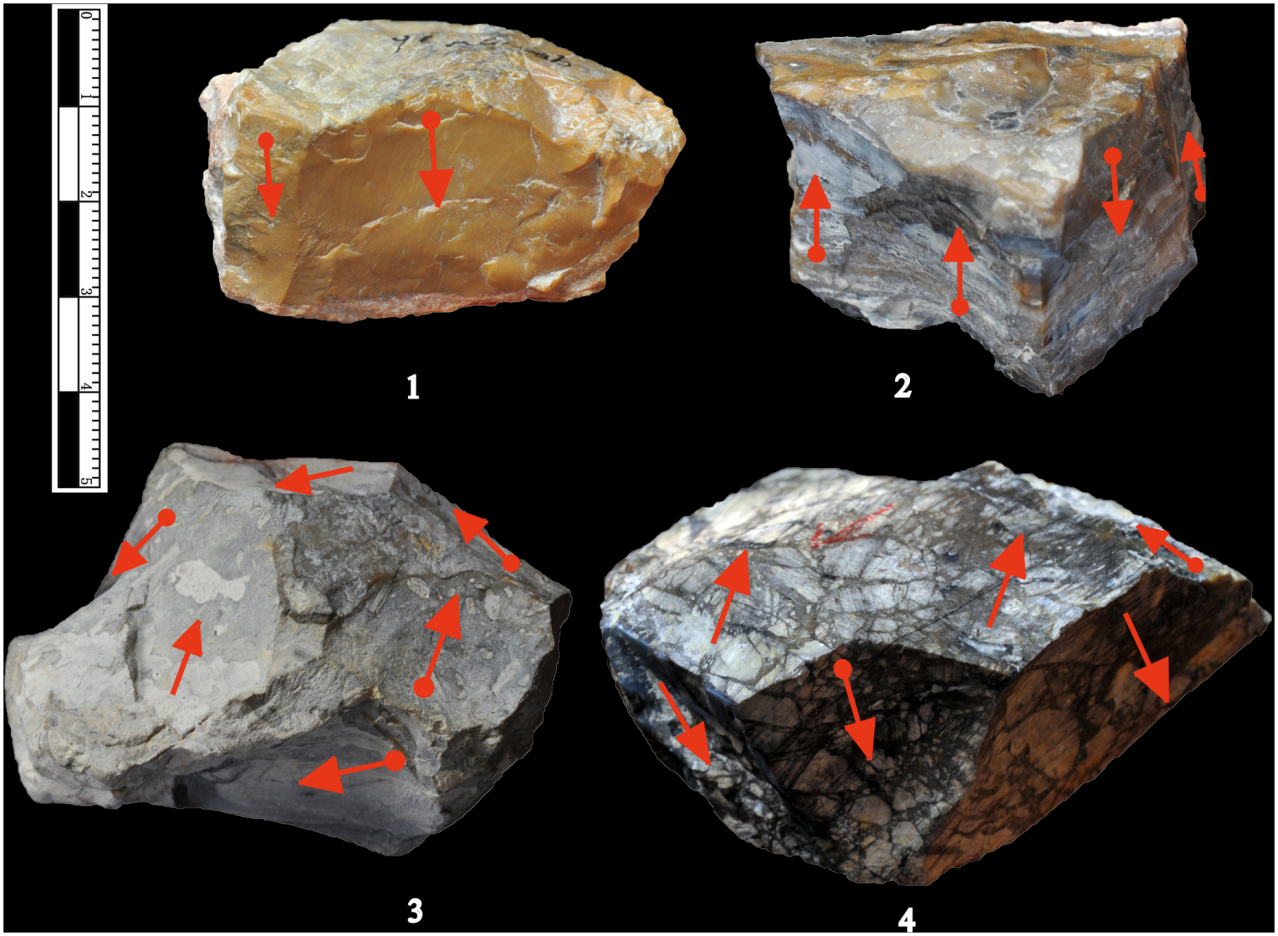
Freehand cores from XCL. Different core types identified from XCL. No. 1 is a Type A core; No. 2 is a Type B core; No. 3 is a Type D core; No. 4 is a Type C core (refer to Fig 4 for types).

**Cores:** The cores have an average maximum length of 50 mm and none exceed 100 mm (Table 2, S1 Appendix). Based on the number and morphology of platforms, the cores were sub-divided into four main types (A-D) (Figs 4 and 5). Type A cores are the most common (n = 25), representing 56% of the total. Type A cores were typically exploited from one direction, generally characterized as a simple flaking method with few prepared platforms only present on three studied cores. Type B cores (n = 7, 17.1%) are comprised of cores that were flaked from two opposite platforms, though with no platform preparation. The Type C cores (n = 6, 14.6%) are bifacially knapped, indicating that the knappers used former flake scars as platforms to produce the follow-on flake. The Type D cores (N = 5, 12.2%) are multidirectional and irregular in flaking. Core surfaces were alternatively flaked through multidirectional removals without a clear organization of the reduction process.

**Table 2:** Lithic types by number and size (mm), subdivided by reduction technique.

Technological system	Main categories	Number	%	Length		Width		Thickness	
				Mean	Std.D	Mean	Std.D	Mean	Std.D
Freehand	Core	43	5.58	52.7	28.8	47.7	23.9	35.2	19.2
	Flake	160	20.75	28.8	13.8	27.9	13.1	10.3	6.6
	Flake frag. and splinter	129	16.73	27.6	9.4	22.6	7.4	10.1	4.3
Bipolar	Core	111	14.40	40.3	17.4	30.7	14.9	19.9	11.3
	Splinter	328	42.52	27.1	9.2	18.1	9.7	8.3	3.8

**Fig 6:**
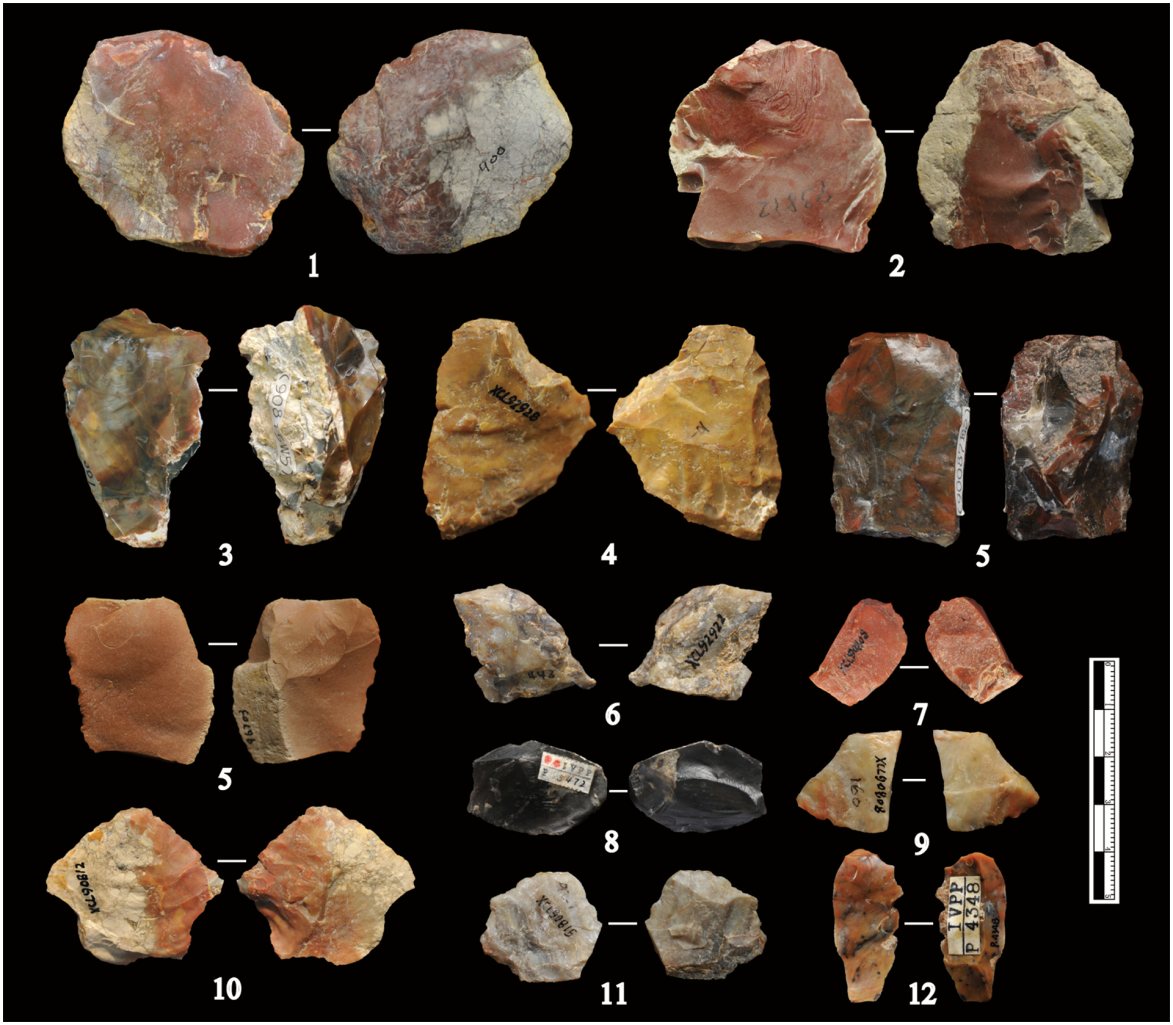
Freehand flakes from XCL. The freehand flakes show Hertzian initiation, i.e. waves, bulbs of force, distinct striking platforms.

**Fig 7:**
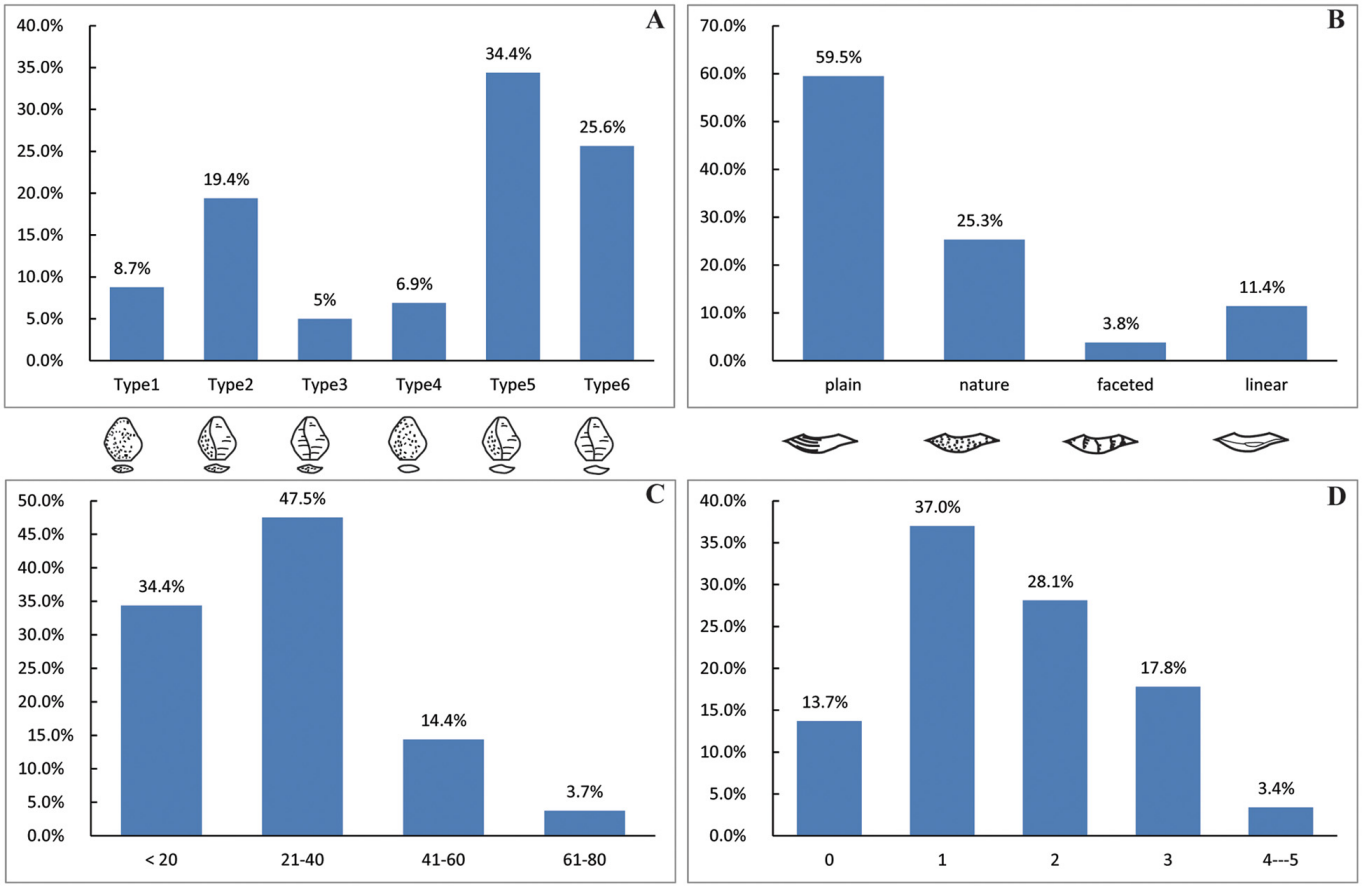
XCL flake attributes. (A) Percentage of cortex on flakes according to Toth’s types [33]; (B) Types of striking platforms on flakes; (C) Flake size ranges (in mm); (D) Number of negative scars on dorsal faces of flakes.

**Flakes:** The flakes have an average maximum length of 27.9 mm (Table 2), and can be generally characterized as small débitage (see Figs 6 and 7). Partitioning flakes by their maximum length indicates that 34.4% average less than 20 mm and 47.5% have a maximum length between 21–40 mm, thereby comprising the majority of the flake assemblage (Fig 7c). The platform angles on flakes are generally steep and range between ca. 70–90°, with a cluster between 80–85°. The flake butts can be divided into four main types: plain (n = 94, 59.49%), natural (n = 40, 25.32%), facetted (n = 6, 3.8%) and linear (n = 18, 11.39%) (Fig 7b). The plain and natural butts account for nearly 85% of the flakes, indicating that freehand percussion was conducted with little evidence for platform preparation. The cortex percentage on dorsal flake surfaces were evaluated according to Toth’s types [31]. The XCL assemblage has a generally high percentage of flakes without cortex (30.63%) (comprising Types 3 and 6 in Fig 7a). Negative flake scar counts are generally low, as 13.7% have no scars and 65.1% have between 1 and 2 scars (Fig 7d). Only 3.4% are well-flaked, having between 4–5 scars. The limited overall number of negative flake scars indicates the low exploitation capacity of the clasts. These quantitative results reinforce previous observations which suggested that cores have little platform preparation and simple exploitation methods.

**Flake fragments:** Flake fragments represent a relatively high proportion of the lithic assemblage, comprising nearly 10% of the total. The ratio of broken to complete flakes is nearly 1:3/4. The broken flakes include “siret” flakes and flakes with broken platforms. Siret flakes are indicative of accidental snapping into two parts along the percussion axis, common in hard hammer percussion [34].

#### Bipolar reduction

In the current study, a large number of bipolar cores and detached pieces (i.e., splinters, broken cores) were identified, accounting for 56.92% of the lithic assemblage (Table 2). The raw materials include chert, quartz and basalt, though chert predominates (as in freehand percussion), representing 93.6% (n = 411) of the total.

**Fig 8:**
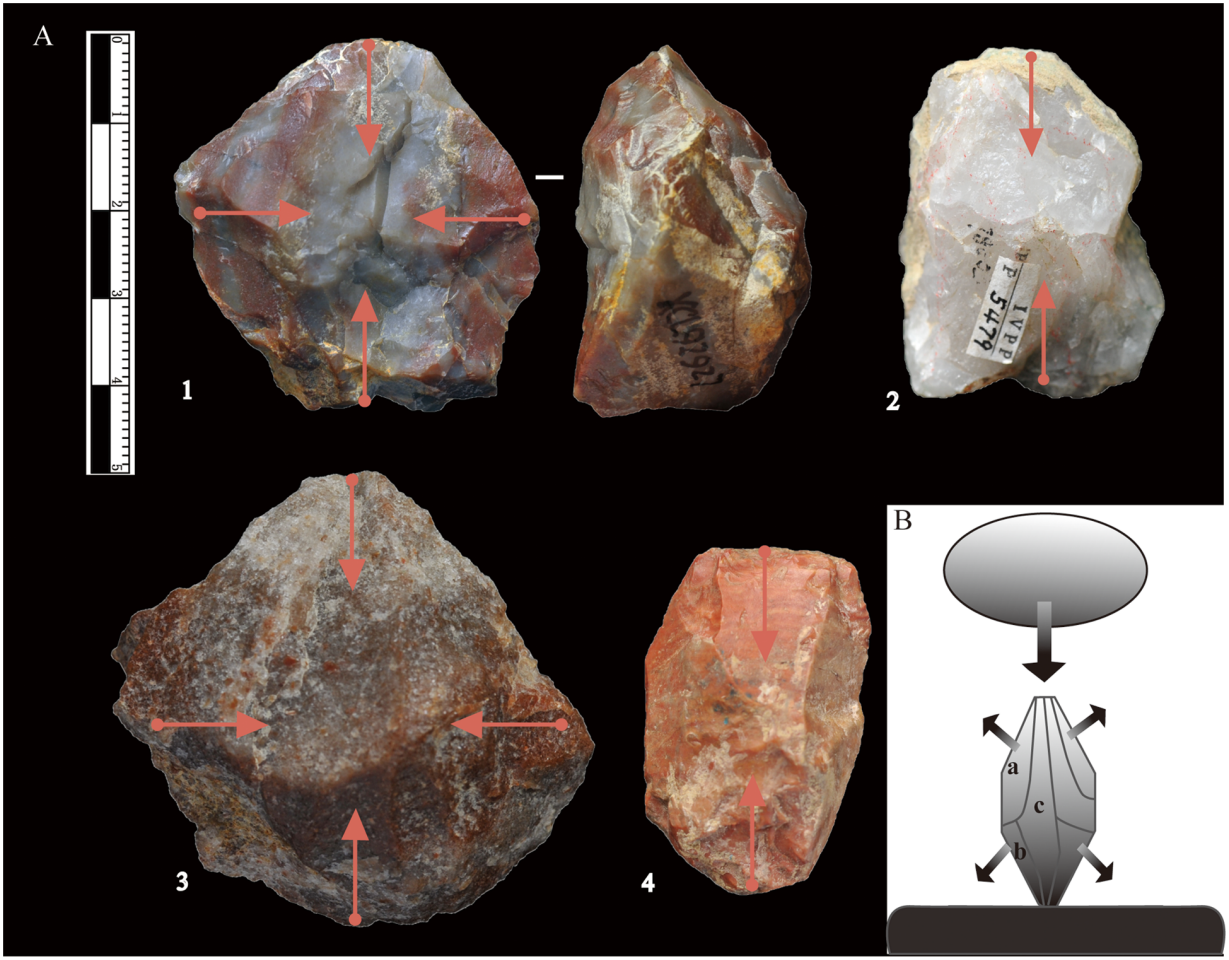
XCL bipolar reduction methods. (A) Bipolar cores, nos. 2 and 4 are striking from a single direction; nos. 1 and 3 are cores with two striking directions; (B) Idealized scheme for bipolar reduction. Note the “a” and ‘b” are pieces showing one observable battering end, and “c” shows two opposite battering ends.

**Bipolar Cores:** The bipolar cores have an average maximum length of 40 mm, slightly smaller than that of freehand cores (Table 2, S1 Appendix). The bipolar cores can be divided into two main groups based on the number of striking directions. The first group (n = 100, 90.1%) includes cores with a stable relationship between the platform and the base, with striking from a single direction (Fig 8A, nos. 2, 4). The second group (n = 11, 9.9%) consists of cores with two striking platforms (see Fig 8A, nos. 1, 3); most of these cores are struck from two perpendicular directions, with a series of platform rotations. The first group can be further sub-divided into two sub-groups according to whether the longest axis is horizontal or vertical to the anvil surface. The results indicate that most of the cores are vertical in battering direction (n = 87), and a few were struck in a horizontal direction. As presented in the idealized scheme in Fig 8B, more enlongated splinters can be obtained when employing a vertical flaking orientation. For clasts with a lack of appropriate striking angles, a horizontal direction may be adopted.

**Fig 9:**
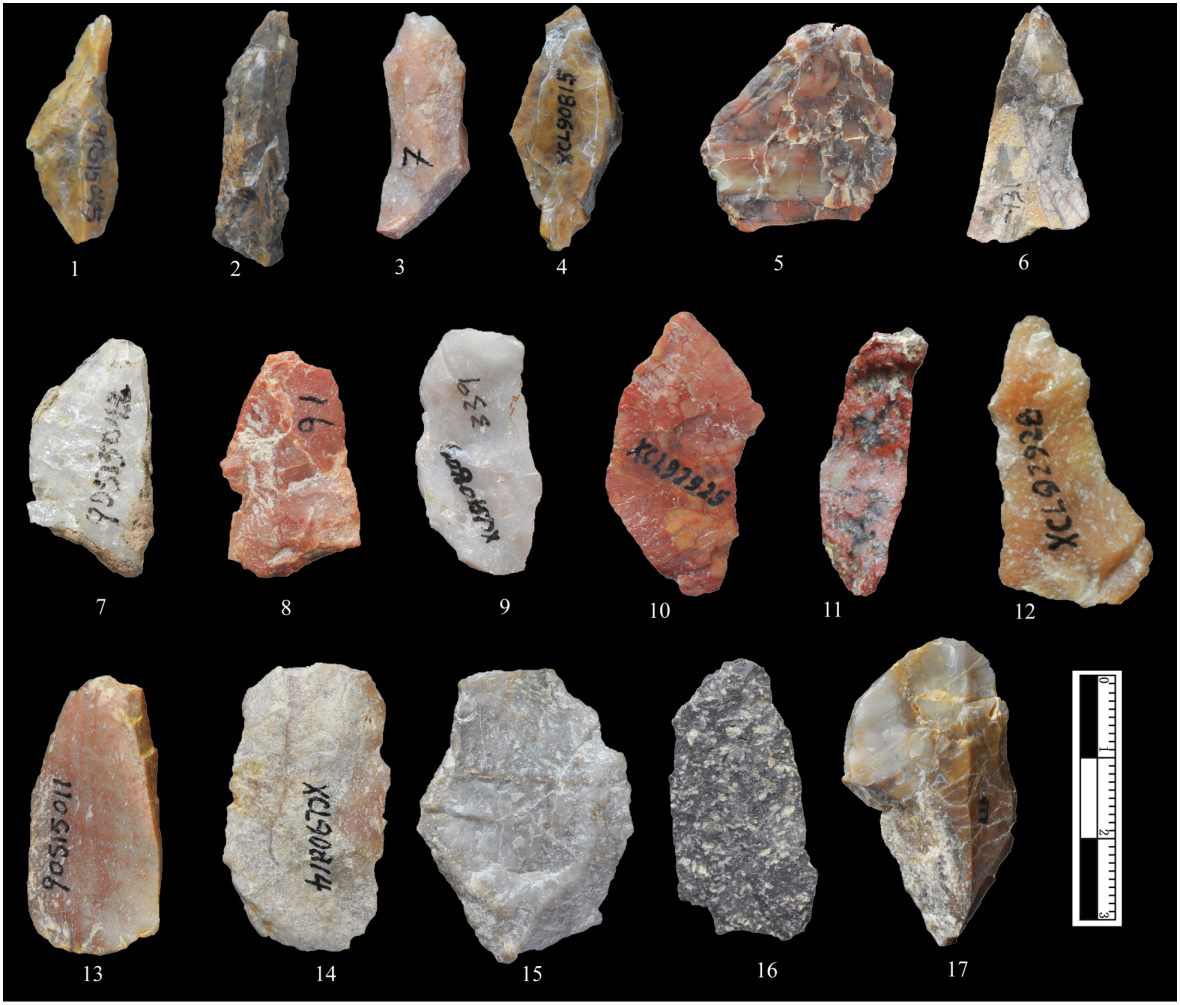
XCL bipolar splinters. The splinters include different raw materials and morphologies. Nos. 7 and 9 are quartz; no. 16 is basalt; all others are chert. Most of the bipolar splinters are small in size and show battering scars on one or two ends.

**Bipolar Splinters:** A large number of bipolar splinters were identified (n = 328) (Fig 9). The pieces have an average maximum length of 27 mm (Table 2), thus somewhat smaller than freehand flakes. Bipolar splinters are defined here as small pieces with crushing on either the platform or the base [35], always without evidence of Hertzian initiation (e.g., bulbs of force, eraillure scars, ripple marks). The bulbs are often diffuse and sometimes disputable. Battering scars can often be observed on two opposite ends and in an axial orientation (Fig 8B-c). Some pieces show one end with obvious battering, whereas the opposite ends appears step or hinge terminations, because they break in the middle of the cores (illustrated in Fig 8B-a and 8B-b).

#### Retouched pieces

**Fig 10:**
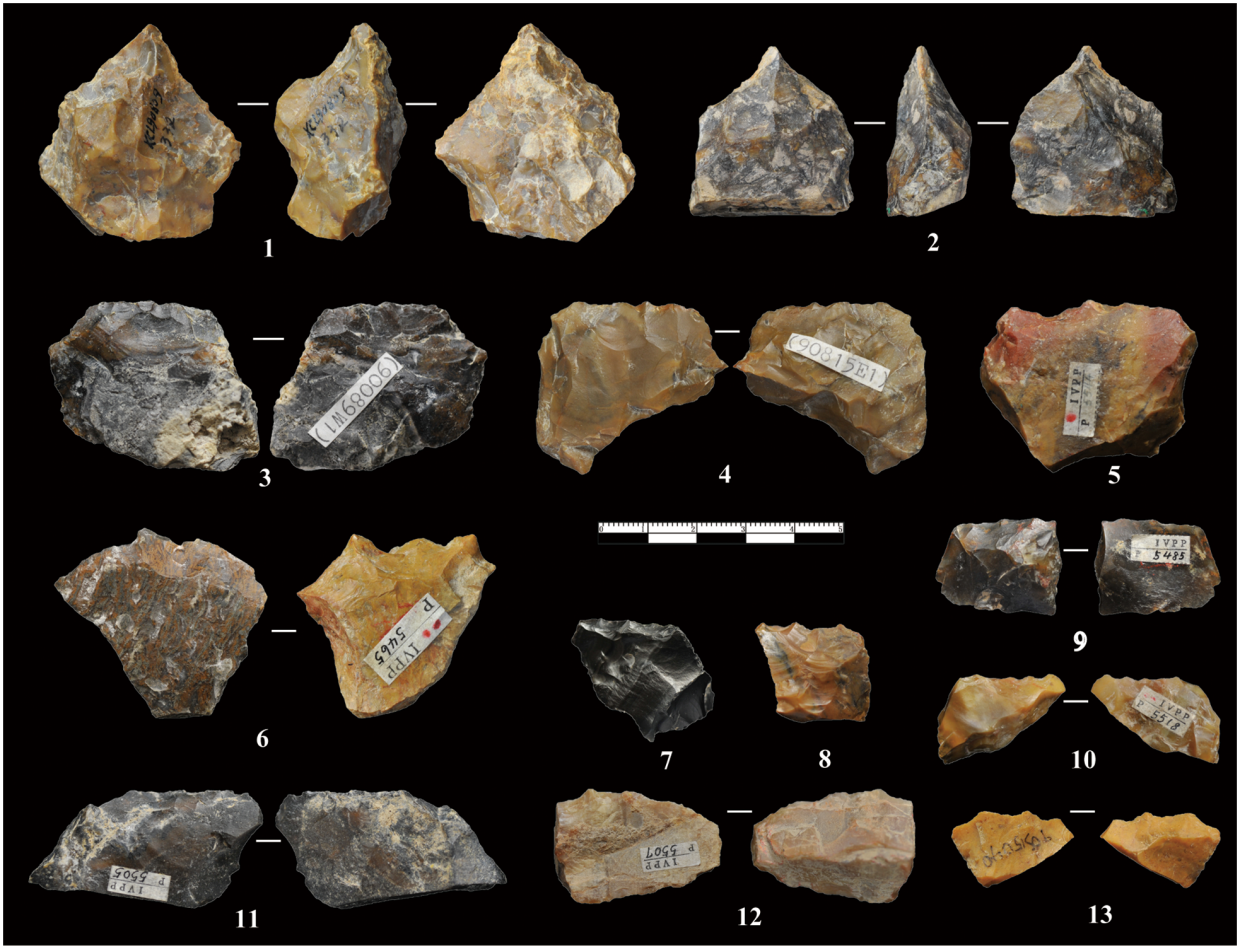
XCL retouched pieces. 1–2: Borers with short retouched points; 3–5, 7–9, 11: Scrapers with continuous retouch along an edge; 6, 10, 12–13: Denticulates showing uneven edges with more than three retouch scars.

A total of 45 retouched pieces was identified (Fig 10, and S2 Appendix), accounting for 3.1% of the lithic assemblage, somewhat higher than reported in previous studies. The retouched pieces are generally small, with an average maximum length of 36.6 mm. In total, 38% of the pieces are retouched either on whole or broken flakes, 13.3% are on bipolar cores or splinters, and others are on angular fragments. On some fine-grained chert pieces, retouching is regular, allowing sub-division into types such as scrapers, denticulates, and borers (Fig 10). Scrapers constitute the largest proportion (71%) of the retouched pieces, defined by three to five continuous retouches on an edge. In these cases, the average length of retouched edges measures 26.86 mm (Fig 10, nos. 3, 4, 9). Two pieces classed as borers are retouched into short points with similar outlines (Fig 10, nos. 1, 2). Though categorized as retouched tools, 13 pieces have no regular patterns. Use-wear studies of the XCL lithics indicated that the retouched tools had no signs of use, whereas some unmodified flakes were used in a variety of motions (e.g., scraping, cutting/sawing, drilling), possibly against plants and animals [19].

## Discussion

### XCL technology in comparison to other Nihewan Basin sites

The lithic findings from XCL can be compared to other Early Pleistocene sites in the Nihewan Basin and to those in other parts of the Old World. Though the Nihewan Basin is known as one of the richest areas in China for Early Pleistocene sites, few lithic studies have been performed on the 15 Lower Palaeolithic sites identified to date. Two exceptions are the lithic analyses conducted at Donggutuo (DGT) [36] and Cenjiawan (CJW) [37], each of which date to ca. 1.1 Ma [38–41].

As shown in Table 3, the three Nihewan Basin sites display evidence of freehand percussion, as illustrated in core technology and flakes. Extensive refitting studies at CJW [41–43], provide some of the best support for the use of freehand percussion in Early Pleistocene industries. In addition, all three sites show evidence for the use of bipolar methods, although in the case of CJW, the number of identified bipolar cores and splinters is very low. In CJW, high numbers and percentages of chunks, shatter and angular fragments may be a further indication that the bipolar method was used, although not identified in previous lithic analysis. Likewise, the relatively high number and percentage of chunks, shatter and angular fragments at DGT may be the consequence of the application of bipolar methods, but not identified in Hou’s [36] earlier comprehensive study. Though not yet fully published, more recent excavations at DGT have indicated the presence of bipolar methods indicated by both cores and flakes [44], consistent with Keates’ [45] observation that rare bipolar pieces could be found in the assemblage. A distinguishing characteristic of DGT is the relatively high number and percentage of freehand cores and flakes, though core technology has been described as unstandardized and non-complex [46, 47]. Hou [48] identified the presence of a more advanced type of wedge-shaped core at DGT, which produced predictably small and elongated flakes, though others [47] have indicated the need to confirm the suggested preparatory flaking strategies. The high incidence of freehand flake morphologies at DGT may possibly relate to the elevated number of finely retouched pieces at the site in comparison to XCL and CJW.

**Table 3:** Comparison of lithic assemblages from XCL, DGT and CJW.

Lithic assemblage	XCL	%	DGT^a^	%	CJW^b^	%
Core (Freehand)	43	2.93	142	9.0	32	2.3
Core (Bipolar)	111	7.56	--	--	4	0.3
Flake (Freehand)	160	11.2	364	23.2	176	12.9
Flake fragment and splinter	129	8.79	--	--	--	--
Splinter (Bipolar)	328	22.34	--	--	2	0.1
Retouched pieces	45	3.07	165	10.5	33	2.4
Unmodified materials	340	19.21	--	---	--	--
Chunk/shatter/angular fragment	370	25.20	900	57.3	1136	83.0
TOTAL	1468	100	1571	100	1368	100

^a^[36]; NB: bipolar cores and flakes have been noted but not yet fully reported (see [44]);

^b^[37]).

On the whole, the comparative analysis suggests that Early Pleistocene hominins in the Nihewan Basin employed various flaking strategies in order to obtain suitable lithic tool assemblages. As has been pointed out, the cherts utilized by the Nihewan hominins are both fine- and coarse-grained, most characterized by internal impurities, joint surfaces and fractures [49]. As has been shown in lithic experimentation, the chert has a tendency to disintegrate during hard hammer percussion [46]. While the poor quality of the raw materials set certain constraints on Nihewan knappers, variable flaking strategies were employed to overcome these limitations. All three sites show the application of freehand and bipolar methods, although bipolar applications appear to be more frequently used at XCL, whereas the freehand technique appears to be used to a much greater degree at DGT and CJW. As has been remarked, it is possible that, in some cases, both methods may have been applied on the same core, i.e., clast could have been first shattered with a hammerstone using direct percussion, and resulting chunks could then be further reduced through bipolar reduction [44]. In some cases, such as at CJW, it has been suggested that hominins intentionally selected some of the best quality cherts [43] in order to employ hard hammer percussion, using both single [41] and multiple platforms [43] to obtain small, useable flakes.

### The XCL technology in wider comparative perspective

To better understand the technological behaviours of Nihewan hominins, it is instructive to compare our findings to well-studied Mode I Pleistocene sites in other geographic regions. At some of the oldest archaeological sites in Africa, at ca. 2.3 Ma, both freehand and bipolar techniques were used, such as at Hadar (A.L. 666) [50] and at Omo [51, 52]. The same is the case for somewhat younger sites, ranging between ca. 1.8 to 1.2 Ma, at Koobi Fora [53–55] and at Olduvai [27, 55]. Outside Africa, freehand percussion and bipolar methods have been identified at sites ranging between ca. 1.4–1.0 Ma, such as at Bizat Ruhama, Israel [56], Fuente Nueva Barranco León, Spain [57], Vallparadís, Spain [58], and at Pont-de-Lavaud, France [29]. In an evaluation of Pleistocene sites across Africa, Europe and Eastern Asia, de Lombera-Hermida and colleagues [29] found that hominins applied variable frequencies and combinations of freehand percussion and bipolar methods (e.g., bipolar-on-anvil, horizontal axial, horizontal non-axial, vertical axial). Bipolar techniques were either complementary or dominant in assemblages and on different blank forms (e.g., pebbles, cobbles, tabular blocks or slabs) and on a range of raw materials (basalt, quartz, quartzite, limestone, chert) of various internal structures and quality. Variability in the frequency of freehand and bipolar methods, and variation in the application of bipolar techniques, provides convincing evidence for considerable technical flexibility on the part of Early and Middle Pleistocene hominins. Such technological versatility allowed early humans to overcome limits of raw materials [29], amply demonstrated by the Nihewan hominins to manufacture suitable tools from some poor quality materials.

The Nihewan lithic assemblages examined here date to between ca. 1.4 and 1.1 Ma, and as far as is currently known, pre-date Large Cutting Tools or Acheulean-like bifacial industries in China by at least 300 ka [59–61]. The appearance of Large Cutting Tool assemblages in China remains the source of current debate, as some authors consider the appearance of handaxes to be the consequence of technological convergence [62] whereas others investigators view the onset of these technologies as a product of both convergence and hominin dispersals from elsewhere [61, 63–67]. Resolving whether handaxe industries were an indigenous development or a product of hominin movements into China, is of interest as archaeologists should expect to find either ‘transitional’ lithic industries or abrupt technological changes. All that can be said with respect to the early Nihewan industries is that they do not provide evidence for an indigenous, transitional development for handaxe industries, as they both pre-date the appearance of bifacial forms and the raw materials available to hominins in the Nihewan basin could not be fashioned into large implements. Despite some technological innovations (e.g., rare prepared cores, blades) in small tool industries in Middle and Late Pleistocene sites in the Nihewan [68, 69], large bifacial forms remain absent in the basin, even though Mode II industries are present ca. 400 km away, in the same valley rift system [70, 71].

## Conclusion

Here we have provided the most up-to-date study of XCL lithic assemblages, a key Early Pleistocene site in the Nihewan Basin of China. At ca. 1.36 Ma, XCL is among the oldest Early Pleistocene sites in China, slightly younger than other Nihewan sites dated to between ca. 1.7–1.6 Ma [2,11]. The presence of hominins in the Nihewan Basin, at 40°N, has been considered a major biogeographic event and a new evolutionary threshold for hominins, as this is the northernmost location of early humans in Eastern Asia [2, 5], though it has been suggested that hominins were not present in these northern latitudes during cold periods or during sub-freezing seasons of the year [72]. During ameliorated periods, Early Pleistocene hominins presumably expanded and utilized the Basin for diet-related activities, implied by the presence of vertebrate fossils in sites and evidence for use-wear on lithics, indicating the processing of plants and animals [19]. Though hominin visits to the Nihewan Basin may have been short term and infrequent, on account of the northern and highly seasonal position of the region [72], our study indicates that Early Pleistocene hominins demonstrated considerable technological flexibility, utilizing freehand and bipolar techniques in variable frequency, thereby overcoming limits of small clast size and poor quality materials to obtain sharp-edged implements for subsistence activities. More widely, the lithic evidence from the Nihewan Basin supports the notion that versatility in Mode I tool-making and behaviour was critical in allowing expanding (and re-expanding) groups of hominins to cope with, and adapt to, unfamiliar Eurasian landscapes and changing habitats [73, 74].

## Supporting Information

S1 Appendix XCL core data base. (PDF)

S2 Appendix XCL retouched tools data base. (PDF)
