# Supplementary material for: The Lithic Assemblages of Xiaochangliang, Nihewan Basin: Implications for Early Pleistocene Hominin Behaviour in North China
Source: PLoS One. 2016 May 20;11(5):e0155793. doi: 10.1371/journal.pone.0155793 (PMC4874576; doi:10.1371/journal.pone.0155793)
Supplement: S1 Appendix — (PDF) [file pone.0155793.s001.pdf]

S1 Appendix. The size and technique of the studied XCL cores

No	Raw material	Tech.	Length	width	thickness
P5470	chert	FHHP	68.9	64.2	33.5
P5469	chert	FHHP	75.7	62.4	44.2
P5472	chert	FHHP	118.5	112.7	80.5
P5468	chert	FHHP	114.8	90.5	60.5
P5488	chert	FHHP	20.1	32.1	27.7
P5506	chert	FHHP	35.4	29.8	16.8
92925	chert	FHHP	30.6	26.2	17.3
90823-132	chert	FHHP	22.9	26	15.4
90812-105	chert	FHHP	32.8	36.8	32.7
93812-2	chert	FHHP	53.4	93.4	42.4
90809-327	chert	FHHP	61.6	45.1	33.1
91810-889	chert	FHHP	108.5	86.4	32.2
90812-1106	chert	FHHP	39	75.4	41.5
90812-(9)	chert	FHHP	76.5	26.8	54.7
90815-54	chert	FHHP	19	26.5	21.9
92920-641	chert	FHHP	46	29.6	21.8
90815-95	chert	FHHP	24.2	36.7	17.3
92928-643	chert	FHHP	45.7	33.5	17.2
90815-73	chert	FHHP	25.9	24.7	9.4
90806	chert	FHHP	99.7	56.5	88.8
92929-273	chert	FHHP	33.6	29.8	29.7
92929	chert	FHHP	30.3	33.1	20.1
90812-(5)	chert	FHHP	31.8	38.8	20.3
92917-(5)	chert	FHHP	24.9	32.3	15.7
90809-332	chert	FHHP	25.8	43.5	41.2
90812-(12)	chert	FHHP	34.8	33.1	21.8
96701+96702	chert	FHHP	43.5	61.5	43.7
90809-68	chert	FHHP	87.5	100.2	88.9
90806-(12)-1	chert	FHHP	45.7	31.22	23.2
90s15016	chert	FHHP	41.4	24.2	27.6
90s15018	chert	FHHP	54.7	61.8	31.9
90s15037	chert	FHHP	49.8	22.9	25.2
90s15047	chert	FHHP	18.3	29.1	16.6
90s15109	chert	FHHP	35.1	28.6	17.2
90s15111	chert	FHHP	34.1	36.1	22.6
92920-641	chert	FHHP	47.4	22.8	29.7
1071	chert	FHHP	27.6	43.7	38.6
90s15295	chert	FHHP	99.4	81.8	44.7

90808-309	chert	FHHP	111.1	72.8	53.4
90815-(18)-1	chert	FHHP	71.1	46.7	35.2
90815-(19)	chert	FHHP	49.3	32.8	49.2
90s15299	chert	FHHP	91.1	69.1	66.2
93812-3	chert	FHHP	56.5	61.5	44
90815	chert	bipolar	35.8	34.1	31.2
92922-(5)	chert	bipolar	17.1	17.9	11.9
P5528	quartz	bipolar	18.4	14.1	8.2
92923-(2)	chert	bipolar	18.4	22.1	10.7
90815E2-237	chert	bipolar	19	17.2	11.8
90815-97	chert	bipolar	19.5	19.4	9.2
92921-408	chert	bipolar	19.8	27.1	16.3
90s15022	quartz	bipolar	20.6	15.8	
90806	chert	bipolar	20.8	16.3	13.8
P5463	quartz	bipolar	20.9	18.3	8.1
90815-96	chert	bipolar	20.9	17.1	12.5
92924-549	chert	bipolar	21.4	29.5	15.9
90815-(2)	chert	bipolar	21.4	15.1	7.7
P5526	chert	bipolar	22.4	16.4	8.3
90810-(20)	chert	bipolar	23.3	18.2	13.4
92925-307	chert	bipolar	23.9	22.9	13.2
P5520	quartz	bipolar	24.7	21.3	13.1
92920-375	chert	bipolar	24.7	15.6	9.1
90s15196	chert	bipolar	25.4	22.8	13.4
P5522	chert	bipolar	26.1	15.1	13.4
P5522	chert	bipolar	26.3	15.1	13.4
92017-(2)	chert	bipolar	26.5	22.2	15.1
90s15021	quartz	bipolar	26.6	18.1	12.1
90805-219	chert	bipolar	26.8	15.5	12.3
90s15044	chert	bipolar	27.1	16.7	11.6
90814	chert	bipolar	27.6	21.2	15.1
92923-534	chert	bipolar	27.8	14.5	8.1
90815-79	chert	bipolar	28.5	15.7	12.5
90814-327	quartz	bipolar	28.6	17.1	16.2
90815-82	chert	bipolar	29.2	18.2	13.4
90s15049	chert	bipolar	29.8	37.4	20.7
90s15092	chert	bipolar	30.1	26.8	17.5
92922-(7)	chert	bipolar	30.2	16.8	12.4
92927-	chert	bipolar	30.4	18.7	15.4
90810-318	chert	bipolar	30.6	29.1	11.1
90806-570	chert	bipolar	30.7	21.2	13.5
90812-349	chert	bipolar	31.1	20.5	17.1
P5541	chert	bipolar	31.4	19.4	7.8

P5513	chert	bipolar	31.5	24.6	13.2
-334	chert	bipolar	31.5	27.6	22.2
92922-(6)	chert	bipolar	31.6	26.1	15.1
90815-48	chert	bipolar	31.8	15.5	12.4
90814	chert	bipolar	32.5	32.9	23.4
90808-151	chert	bipolar	32.9	21.2	15.7
90810w1	quartz	bipolar	33.1	27.7	21.4
93812-11	chert	bipolar	33.1	27.5	11.1
92922-(4)	chert	bipolar	33.3	29.7	18.4
97702-(1)	chert	bipolar	33.4	24.9	13.1
90815-366	chert	bipolar	33.5	27.2	26.2
92922-121	chert	bipolar	34.1	25.5	12.2
90809-(11)	chert	bipolar	34.1	21.9	14.7
90s15260	chert	bipolar	34.4	31.6	20.1
90815-(8)	chert	bipolar	34.6	25.4	15.2
90814-(33)	chert	bipolar	35.1	19.5	12.9
90814-1010	chert	bipolar	35.8	36.1	25.3
90815-1065	chert	bipolar	36.2	37.1	25.9
90812-(17)	chert	bipolar	36.3	31.6	22.1
P5479	quartz	bipolar	38	26.2	17.9
92927-4	chert	bipolar	38.4	36.7	22.2
-340	chert	bipolar	38.6	28.9	7.9
90808-593	chert	bipolar	38.7	23.7	14.5
90808-141	chert	bipolar	38.8	23.8	16.3
-914	chert	bipolar	39.4	37.1	26.3
92921-919	chert	bipolar	40.1	24.2	11.3
92923-435	chert	bipolar	40.6	16.7	12.7
90s15112	chert	bipolar	40.7	37.7	18.6
90808-(33)	chert	bipolar	40.9	22.2	17.7
90816-(12)	chert	bipolar	41.1	26.7	18.1
90806-576	chert	bipolar	41.5	21.7	16.5
-411	chert	bipolar	42.6	8.4	15.7
-707	basalt	bipolar	42.9	24.8	15.1
90087W2	chert	bipolar	43.2	48.2	12.6
90s15017	chert	bippolar	43.4	69.3	21.9
97701	chert	bipoalr	43.9	41.8	40.9
92929-(6)	chert	bipolar	44.5	27.8	16.9
90810-696	quartz	bipolar	45.4	25.3	25.2
90809-131	chert	bipolar	45.6	51.1	21.4
90808-(31)	chert	bippolar	46.7	25.2	18.1
92925-(3)	chert	bipolar	46.9	28.9	22.8
92705-	chert	bipolar	47.2	18.9	11.4
90814-(22)	chert	bipolar	47.2	27.7	20.3

90s15136	basalt	bipolar	47.7	28.8	16.1
90814-348	chert	bipolar	48.2	43.8	21.7
92917-(11)	chert	bipolar	48.3	26.1	15.4
90808-(22)	chert	bipolar	48.4	19.9	15.6
90814-(12)	chert	bipolar	48.6	27.6	18.1
90809-326	chert	bipolar	49.1	47.2	43.7
497	chert	bipolar	50.2	60.5	44.1
90809-830	chert	bipolar	50.8	47.8	31.3
90808-(26)	chert	bipolar	50.8	47.3	17.1
90806-195	chert	bipolar	51.1	48.4	17.8
90810-498	chert	bipolar	52.8	46.2	38.4
90808-?	chert	bipolar	53.2	42.2	24.5
90810-699	chert	bipolar	53.3	26.8	25.9
90814-1013	chert	bipolar	57.1	49.1	17.6
90814-(55)	chert	bipolar	59.9	45.7	28.3
92929-(12)	chert	bipolar	60.3	38.7	31.1
90815-(20)	chert	bipolar	61.7	41.3	29.6
90808-580	chert	bipolar	62.1	59.1	35.9
90814-(48)	chert	bipolar	63.1	34.8	30.1
90814-1012	chert	bipolar	66.9	53.1	41.7
92925-1016	quartz	bipolar	70.4	73.7	43.3
90815-(15)	chert	bipoalr	70.8	53.1	36.1
92925-1016	quartz	bipolar	71.8	74.8	54.4
90815-(16)	chert	bipolar	72.3	51.9	29.8
90808-(48)	chert	bipolar	75.4	59.5	31.4
90814-1021	chert	bipolar	77.4	53.1	36.1
90808-(50)	basalt	bipolar	77.4	39.5	22.7
90s15297	chert	bipolar	79.1	56.9	36.2
90814-(49)	chert	bipolar	82.1	30.4	28.2
90805	chert	bipolar	119.1	85.1	60.6
