# Supplementary material for: The Lithic Assemblages of Xiaochangliang, Nihewan Basin: Implications for Early Pleistocene Hominin Behaviour in North China
Source: PLoS One. 2016 May 20;11(5):e0155793. doi: 10.1371/journal.pone.0155793 (PMC4874576; doi:10.1371/journal.pone.0155793)
Supplement: S2 Appendix — (PDF) [file pone.0155793.s002.pdf]

S2 Appendix. The size (mm) and retouch features of the studied XCL tools

No	Type	Raw material	blank	Length	Width	Thickness	funcation length	funcation depth	retouch scar
P5473	Notch	Chert	bipolar core	32.8	25.6	9.3			
P5471	scraper	chert	block	43.9	46	15.5	28.8	7.3	
P5503	borer	chert	block	34.3	34	18.9	6.5		
P5518	scraper	chert	block	28.1	14.9	7.8	13.6	8.2	3
P5509	scraper	chert	block	35.7	19.6	11.1	19.3	3.2	
P5510	denticulate	chert	broken flake	22.4	21.2	11.2	19.2		
P5505	scraper	chert	broken flake	44.1	23.5	7.7	22.5		5
P5501	scraper	chert	flake	31.1	27.4	13.9	19.6	5.9	3
P5511	scraper	chert	bipolar core	37.1	26.7	12.2	13.9		
P5546	scraper	Chert	broken flake	45.8	25.5	11.5	23.3		
P5544	scraper	chert	flake	40.5	32.4	14.7	40.5	8.6	5 to 6
P5553	scraper	chert	block	37.1	30.2	15.2	24.9	9	5
P5466	scraper	chert	block	51.5	30.8	35.9	30.2+22.8		
P5507	scraper	chert	block	34	23.4	11.4	30.3+22.6	4.9	
P5515	scraper	chert	flake	32.7	19.3	4.5	10.4+14.5+15.2		
P5464	scraper	chert	flake	47.3	42.8	17.7	29.9	7.3	5
P5497	scraper	Chert	flake	43	36.2	16.1	38.9(L)17.4+18.4(R)		10?
P5496	scraper	chert	flake	43.9	22.4	18.6	41.9+34.6	8.8	
P5474	dendiculate	chert	flake	48.6	37.2	11.3	43.2		6
P5534	scraper	chert	block	32.4	30.9	18.8	29.4		
P5465	denticulate	chert	broken flake	39.7	36.3	13.9	36.7	10.1	4

P5491	scraper	chert	?	63.3	35.5	20.6	30.3	4.9	
P5495	borerr	chert	block	45.1	36.7	12.7	2.9		
90815-207	borer	chert	block	21.7	22	12	5.9	9/8.9	2
90816W3-178	scraper	chert	broken core	27.1	21.5	13.3	13.2	1.7	
90810-701	scraper	chert	block	48.1	31.6	18.6	26.1	10.1	
92927	scraper	chert	flake fragment	34.8	27.7	7.3	22	6	4
90806	unidentified	chert	spliner(B)	13.4	13.1	11.4			
90816-	unidentified	basalt	spliner(B)	32.6	14.7	8.8			
90805-253	scraper	chert	flake	30.7	16.8	12.5			
90812	unidentified	chert	spliner(B)	35.7	29.2	13.1			
90809-365	scarper	basalt	block	53.1	42.9	21.3	33.9	7.3	
90812-(1)	scraper	chert	splint pebble	54.4	48.5	23.2	26.2	5.1	6
90812-(2)	scraper	chert	flake fragment	19.5	20.2	8.9	22+13.8	6.6	
90089W1	scraper	chert	flake fragment	46.5	34.8	9.7	25.7	8.6	
90815E 1	scraper	chert	flake	42.2	29.6	12.1	34.7	10.1	
90085W2	scraper	chert	flake	36.8	40.6	13.5	39.7	10.4	5
90s15040	scraper	chert	Spliner(B)	24.3	16.7	6.5	22.7	7.8	
90s15073	scarper	chert	flake fragemnt	27.7	19.7	11.9	18.3	9.2	
90s15235	Notch	chert	fragment	11.1	9.9	4.4			
90s15236	scraper	chert	fragment	30.5	20.9	15.4	18.4	4.7	
90814-(32)	Notch	Chert	bipolar core	27.1	25.9	13.9			
90816-(18)	scraper	chert	splinter（B)	23.5	11.3	6.7	22.8	6.6	
90808-(52)	scraper	chert	fragment	52.8	37.8	17.9	20.1	9.4	
90815-(21)	Notch	chert	fragment	38.9	39.8	22.3			
